# Surgical Management of Juxtaductal Coarctation of the Aorta in Adults: A Novel Technique

**DOI:** 10.7759/cureus.66843

**Published:** 2024-08-14

**Authors:** Arun Sharma, Rupesh Kumar, Vidur Bansal, Sunder Negi, Hemant Kumar

**Affiliations:** 1 Radiology, Postgraduate Institute of Medical Education and Research (PGIMER), Chandigarh, IND; 2 Cardiothoracic and Vascular Surgery, Postgraduate Institute of Medical Education and Research (PGIMER), Chandigarh, IND; 3 Cardiothoracic and Vascular Anesthesiology, Postgraduate Institute of Medical Education and Research (PGIMER), Chandigarh, IND; 4 Cardiothoracic and Vascular Surgery Intensive Care Unit, Postgraduate Institute of Medical Education and Research (PGIMER), Chandigarh, IND

**Keywords:** aortic anomaly, juxtraductal aotra, adult coarctation, coarctation, bypass, extra-anatomical

## Abstract

Coarctation of the aorta (CoA) is a rare congenital malformation, the symptoms of which may remain subtle in childhood and appear at a later age. It can manifest only with symptoms of upper body hypertension. Various methods have been described for managing coarctation of the aorta in adults, including surgical or percutaneous balloon angioplasty with or without stent placement and medical therapy.

Surgical approaches include an extra-anatomical bypass through a left lateral thoracotomy, a median sternotomy, or a combined median sternotomy and a laparotomy incision; all have their merit in overcoming the symptoms. We went ahead with an extra-anatomical tube graft between the ascending aorta and the descending thoracic aorta in a 24-year-old patient who presented to us with a diagnosis of coarctation of the aorta.

## Introduction

Coarctation of the aorta (CoA) is a rare congenital cardiac malformation that can go undiagnosed until old age. It can manifest only with symptoms of upper body hypertension, and other clinical signs can be subtle and overlooked if a complete physical exam is not performed. Various methods have been described for the management of coarctation of the aorta in adults, including surgical or percutaneous balloon angioplasty with or without stent placement and medical therapy.

Surgical techniques include an extra-anatomical bypass through a left lateral thoracotomy, a median sternotomy or a combined median sternotomy, and a laparotomy incision. We went ahead with an extra-anatomical tube graft between the ascending aorta and the descending thoracic aorta in a 24-year-old patient who presented with a diagnosis of coarctation of the aorta.

## Case presentation

**Figure 1:**
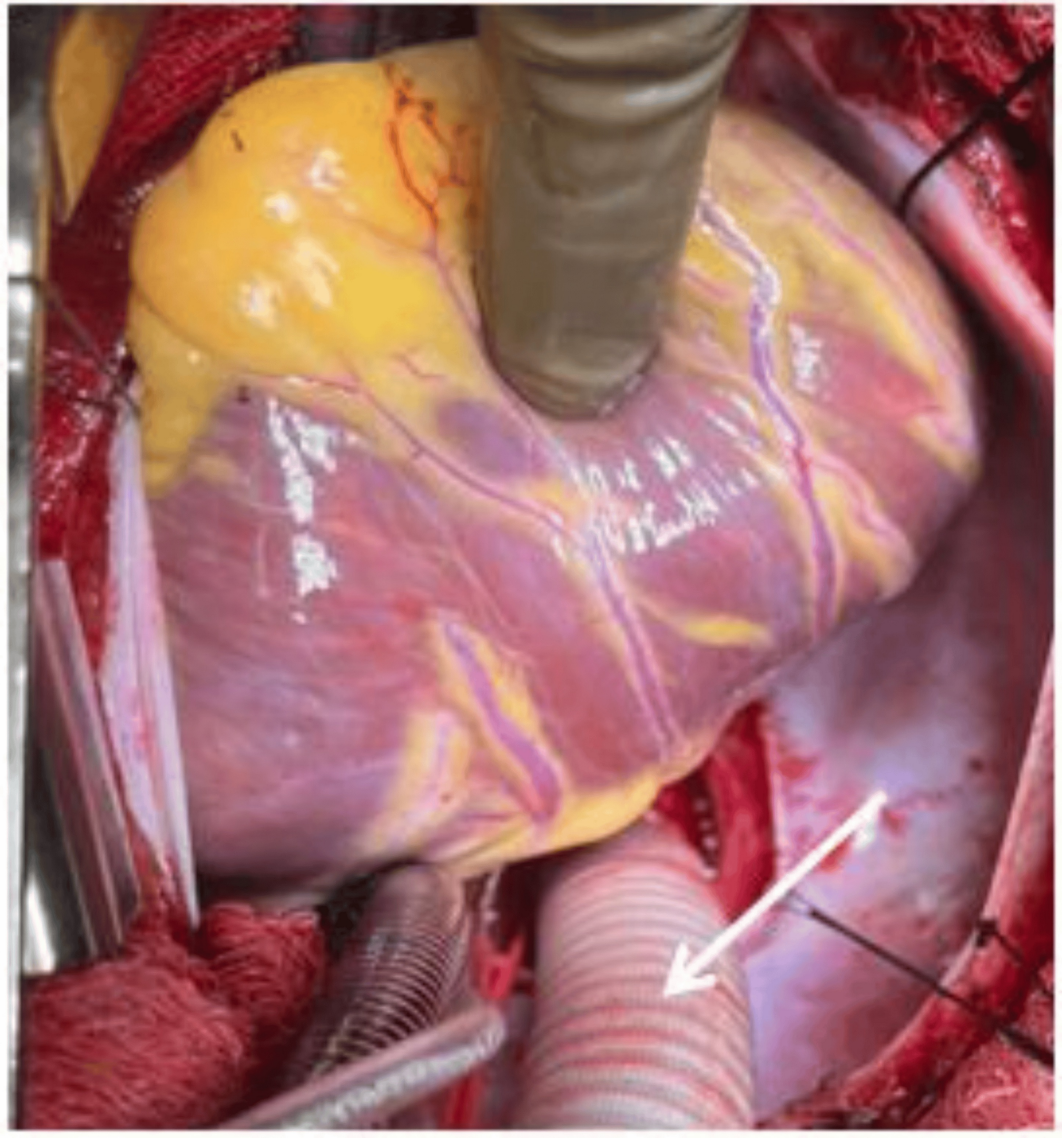
An operative picture showing 16 mm Dacron tube graft (arrow) anastomosis to the descending thoracic aorta near the hiatus in an end-side manner

**Figure 2:**
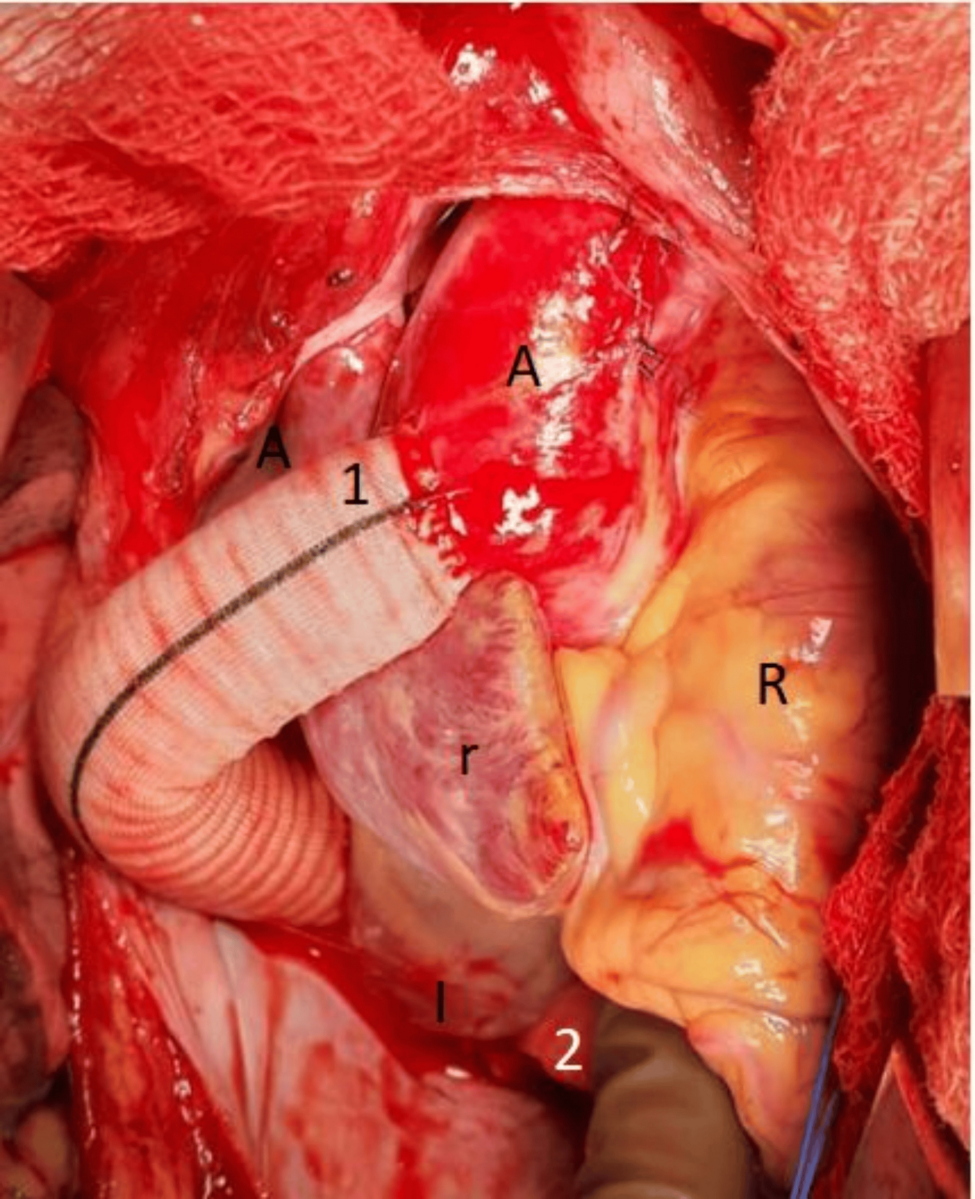
An operative picture showing anastomosis of graft to the ascending aorta in an end-side manner coursing the graft behind the inferior vena cava A: ascending aorta; I: inferior vena cava; R: right ventricle; r: right atrium; 1: ascending aorta to Dacron tube graft anastomosis; 2: Dacron tube graft to descending thoracic aorta anastomosis

A 24-year-old patient presented to the outpatient department of our hospital with complaints of headache and dyspnea. On physical examination, he was found to have a systolic gradient of 44 mmHg between the right upper limb and the left lower limb. The patient further underwent a trans-thoracic echocardiography, which showed a discrete narrowing in the descending thoracic aorta just distal to the take-off of the left subclavian artery with a gradient of 75 mm of Hg across it. The patient underwent a CT angiogram to confirm the diagnosis and plan a definitive surgical repair. After a detailed discussion in a heart team meeting, we decided to go ahead with an extra-anatomical ventral aorta repair via a median sternotomy. After initiating cardiopulmonary bypass, the ascending aorta was cross-clamped, cardiac electromechanical quiescence was achieved by administration of St. Thomas II antegrade cardioplegia at the rate of 20 ml/kg every twenty minutes, and antegrade flow was started through the ascending aorta. A 16 mm Dacron tube graft (Intergard Woven, Maquet) was anastomosed to the descending thoracic aorta near the hiatus in an end-side manner (Figure 1), followed by an anastomosis to the ascending aorta in an end-side manner (Figure 2) by passing the graft behind the inferior vena cava.

**Figure 3:**
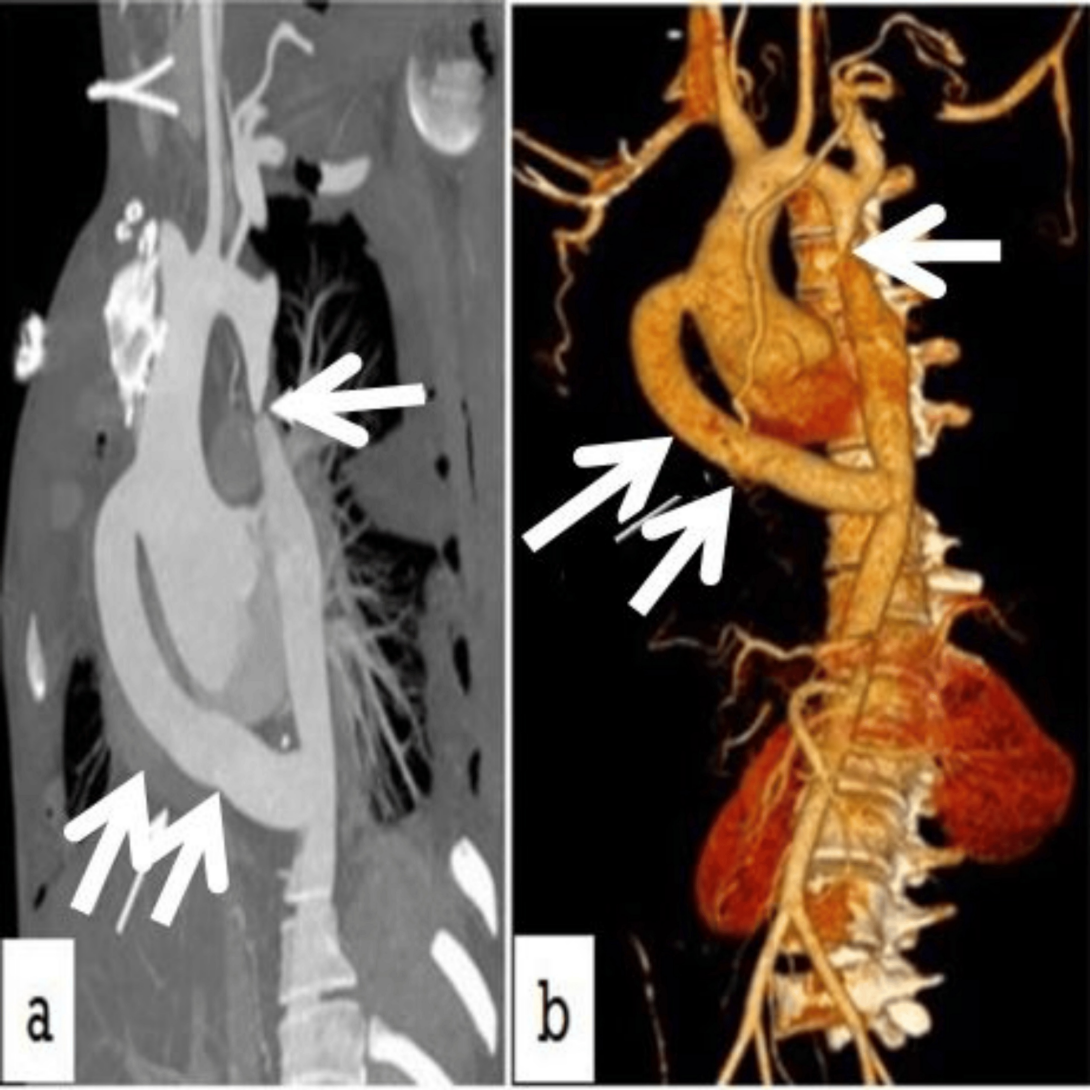
CT-aortogram findings a: postoperative CT-aortogram showing a well-opacified tube graft from the ascending aorta to the descending aorta with the native coarctation in situ (single arrow: juxtraductal segment of coarctation of the aorta; double arrows: Dacron tube graft); b: post-procedure three-dimensional reconstruction of CT aortogram (single arrow: juxtraductal segment of coarctation of the aorta; double arrows: Dacron tube graft)

The immediate postoperative gradient between the right radial and femoral arteries was 1 mm of Hg, accepted as the success of the procedure. Postoperatively, the patient was extubated on postoperative day one and was discharged on postoperative day five. The patient is currently doing well in the postoperative period. A CT-aortogram was done at six weeks postoperatively (Figure 3a, 3b), which showed a well-opacified tube graft from the ascending aorta to the descending aorta with the native coarctation in situ.

## Discussion

Coarctation of the aorta (CoA) is a rare congenital cardiac malformation that can go undiagnosed until old age. It sometimes manifests only with symptoms of upper body hypertension, and other clinical signs can be subtle and overlooked if a complete physical exam is not performed [1]. Surgical techniques include an extra-anatomical bypass through a left lateral thoracotomy, a median sternotomy or a combined median sternotomy, and a laparotomy incision [2]. The incidence of this disease is approximately 5-8% of all congenital heart disease [3]. It is a surgical emergency in neonates with refractory heart failure, whereas adults with a long-standing coarctation can present with refractory hypertension and extensive collateral vessels. In adults, CoA repair may require extensive dissection and deep hypothermic circulatory arrest, which poses a risk of cerebrovascular complications to an individual. Opening the posterior pericardium behind the phrenic nerve gives excellent exposure to the descending thoracic aorta at the hiatus, and passing the bypass graft behind the inferior vena cava with or without cardiopulmonary bypass is an attractive surgical approach [4]. The extra-anatomical bypass avoids the dissection of extensive collateral circulation, as typically seen in adults [5]. The extra-anatomic approach avoids extensive aortic mobilization and dissection near the neurovascular structures. Intervention for coarctation in patients can be achieved by bypass graft across the area of coarctation when end-to-end anastomosis seems unfeasible [6]. The procedure can be performed via sternotomy, enabling concomitant intracardiac and/or proximal aortic repair. It is very important to understand the surgical steps, like firstly anastomosing the tube graft with the supra-diaphragmatic segment of the descending thoracic aorta in an end-to-side fashion, followed by coursing the other end of the tube graft behind the inferior vena cava, as it avoids the compression of the inferior vena cava and hence the impendence of venous return to the right atrium. The final procedure ends with sizing the proper length of the tube graft by distending it with saline or filling it with blood flow, followed by the end-to-side anastomosis on the right lateral aspect of the ascending aorta. There is a role of biophysics here, as the anastomosis over the ascending aorta should not be much below near the sinuses, as there may be kinking of the tube graft, and the right atrium may also get compressed; moreover, there is a probability of carotid steal if the anastomosis is done much below, near the aortic sinuses. Hence the anastomosis should be done as near as the origin of the innominate artery. There is another mathematical calculation of the size of ostia creation over the aorta for the anastomosis. The length of the aortotomy for anastomosis should match the diameter of the descending thoracic aorta near the hiatus. Too big anastomosis will lead to flattening of the aorta-graft anastomosis and a risk of carotid steal, whereas too small anastomosis will leave the procedure with a residual gradient. The gradient between the right radial and femoral arteries should always be checked on the operation table, and a single-digit gradient between these two vessels should be accepted as the success of the procedure. Both interrupted and continuous suturing techniques with a non-absorbable suture are feasible, but we did a continuous suturing of the anastomosis with a 4-0 polypropylene suture at both ends as it is a little fast to complete the procedure. The procedure of extra-anatomic aortic bypass is an attractive treatment option for complex aortic coarctation with low morbidity and mortality, and moreover, the midterm results are also favorable [7]. The ascending-descending aortic bypass through a posterior pericardial approach is safe in relieving the symptoms due to the above pathology [8]. Though the primary repair of coarctation in adults is effective for the elimination of stenosis in simpler cases, it also mandates a close follow-up due to the risk of re-coarctation and necessitates a close follow-up after repair [9]. There are various approaches to performing an extra-anatomic aortic bypass, like median sternotomy or median sternotomy-laparotomy; more ever, it is a preferable single-stage approach for patients with concomitant complex coarctation and cardiovascular disorders, and hence this approach of median sternotomy was applied in our case [10].

## Conclusions

The extra-anatomic approach is a feasible approach in adults with coarctation of the aorta, as it avoids an extensive dissection of the segment of relatively non-pliable aorta near the juxtraductal coarctation and minimizes the risk of injury to the collateral vessels and recurrent laryngeal nerve. This technique effectively relieves obstruction, much similar to the end-to-end repair techniques in most neonates and children.

## References

[1] Yin K, Zhang Z, Lin Y (2017). Surgical management of aortic coarctation in adolescents and adults. Interact Cardiovasc Thorac Surg.

[2] Mishra AK, Barwad P, Bansal V, Mandal B, Srivastava A, Naganur SH (2020). Ebstein's anomaly of tricuspid valve with aortic stenosis and coarctation of aorta: successful single-stage repair of a rare adult congenital heart disease. J Card Surg.

[3] Baumgartner H, De Backer J, Babu-Narayan SV (2021). 2020 ESC Guidelines for the management of adult congenital heart disease. Eur Heart J.

[4] McKellar SH, Schaff HV, Dearani JA (2007). Intermediate-term results of ascending-descending posterior pericardial bypass of complex aortic coarctation. J Thorac Cardiovasc Surg.

[5] Feins EN, Jassar AS, Tapias LF, Isselbacher EM, Sundt TM 3rd (2018). Extraanatomic bypass of a complex adult coarctation. Ann Thorac Surg.

[6] Benomrane S, Soumer K, Khayati A (2015). Adolescent coarctation of aorta treated with subclavian-descending aorta bypass grafting. Cardiol Young.

[7] Almeida de Oliveira S, Lisboa LA, Dallan LA, Abreu F CA, Rochitte CE, de Souza JM (2003). Extraanatomic aortic bypass for repair of aortic arch coarctation via sternotomy: midterm clinical and magnetic resonance imaging results. Ann Thorac Surg.

[8] Zhang T, Juan C, Yu WC, Zhang HZ, Zou CW (2020). Rare complex coarctation of aorta: treated with extra-anatomic aortic bypass approach. Circ Cardiovasc Imaging.

[9] Egbe AC, Miranda WR, Warnes CA, Bonnichsen C, Crestanello J, Anderson JH, Connolly HM (2021). Persistent hypertension and left ventricular hypertrophy after repair of native coarctation of aorta in adults. Hypertension.

[10] Wang R, Sun LZ, Hu XP (2010). Treatment of complex coarctation and coarctation with cardiac lesions using extra-anatomic aortic bypass. J Vasc Surg.

